# Kaposi sarcoma with extensive thoracic involvement

**DOI:** 10.1590/0037-8682-0598-2025

**Published:** 2026-03-30

**Authors:** Antônio Carlos Portugal Gomes, Bruno Hochhegger, Alexandre Dias Mançano, Gláucia Zanetti, Edson Marchiori

**Affiliations:** 1 Medimagem/BP Medicina Diagnóstica, São Paulo, SP, Brazil.; 2 University of Florida, Department of Radiology, Gainesville, FL, USA.; 3 Universidade Federal do Rio de Janeiro, Departamento de Radiologia, Rio de Janeiro, RJ, Brasil.

**FIGURE 1:**
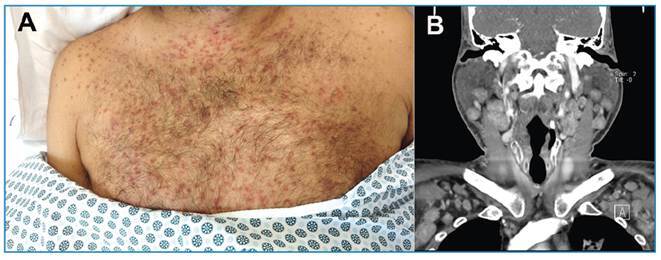
**A.** The upper trunk showing disseminated red and purple skin lesions. **B.** Computed tomography image of the cervical region showing bilateral and relatively symmetrical lymphadenopathy, affecting mainly the cervical and supraclavicular chains.

**FIGURE 2:**
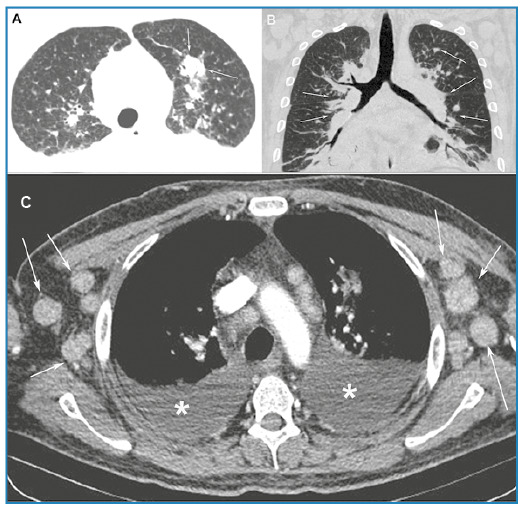
Computed tomography (CT) images of the chest with axial (**A**) and coronal (**B**) reconstructions showing an irregular parenchymal nodule (arrows in **A**) and thickening of the peribronchovascular interstitium and interlobular septa (arrows in **B**). **C.** Axial CT image showing axillary lymph-node enlargement (arrows) and bilateral pleural effusions (asterisks).

A 45-year-old male diagnosed with acquired immunodeficiency syndrome (AIDS) who was irregularly receiving antiretroviral therapy, reported episodes of fever and general malaise for the previous 30 days, followed by the appearance of disseminated red and purple skin lesions, predominantly on the upper trunk (Figure 1A). Computed tomography (CT) of the neck revealed bilateral lymphadenopathy, mainly affecting the cervical, supraclavicular, and axillary chains (Figure 1B). The CD4 cell count was 145 cells/mm^3^ and the viral load was 46,000 copies/mL. Panel results for other sexually transmitted diseases were unremarkable. Subsequently, the patient developed dyspnea and a dry cough. CT of the chest revealed irregular thickening of the peribronchovascular interstitium and interlobular septa, parenchymal nodules, mediastinal and axillary lymph node enlargement, and bilateral pleural effusion (Figure 2A-C). Flexible bronchoscopy revealed multiple lesions in the tracheobronchial mucosa, consistent with Kaposi sarcoma (KS). An axillary lymph node biopsy was also diagnostic of KS. Due to complications, the patient died three months later.

Kaposi sarcoma is a malignant angioproliferative mesenchymal tumor caused by human herpes virus-8. AIDS-related KS is aggressive and frequently fatal. Pulmonary manifestations can involve the tracheobronchial tree, parenchyma, and pleural space. The most frequent CT findings in AIDS-related KS include peribronchovascular and interlobular septal thickening, ill-defined parenchymal nodules, fissural nodules, lymph node enlargement, and pleural effusion. The incidence of KS in the population with AIDS has dramatically decreased in this era of highly active antiretroviral therapy^1^ ^-^ ^4^.

In conclusion, KS should be included in the differential diagnosis of disseminated lymphadenopathy-associated skin lesions in patients with AIDS. 
